# Unstable Jobs Cannot Cultivate Good Organizational Citizens: The Sequential Mediating Role of Organizational Trust and Identification

**DOI:** 10.3390/ijerph16071102

**Published:** 2019-03-27

**Authors:** Byung-Jik Kim

**Affiliations:** 1 Sogang Business School, Sogang University, Seoul 04107, Korea; kimbj82@business.kaist.edu; Tel.: +82-2-958-3624; 2 College of Business, Korea Advanced Institute of Science and Technology, Seoul 02455, Korea

**Keywords:** job insecurity, organizational citizenship behavior, organizational trust, organizational identification, sequential mediation model

## Abstract

Although existing works have investigated the influence of employee’s job insecurity on his or her perceptions or attitudes, those studies relatively have paid less attention to the influence of it on employee’s behaviors, as well as to its intermediating mechanisms of the relationship between job insecurity and the behaviors. Considering that employee’s behaviors substantially influence various organizational outcomes, I believe that studies which examine the impact of job insecurity on the behaviors as well as its underlying processes are required. Grounded on the context–attitude–behavior framework, I delved into the intermediating mechanism between job insecurity and organizational citizenship behavior with a sequential mediation model. In specific, I hypothesized that employee’s organizational trust and organizational identification would sequentially mediate the job insecurity–organizational citizenship behavior (OCB) link. Utilizing 3-wave time-lagged data from 303 employees in South Korea, I found that organizational trust and organizational identification function as sequential mediators in the link. The finding suggests that organizational trust and organizational identification are underlying processes to elaborately explain the job insecurity–OCB link.

## 1. Introduction

As global competition and uncertainty have been intense, many firms are experiencing considerable pressures to maximize the efficiency of its operation. To achieve the inevitable goal, those companies have increased job instability through downsizing, outsourcing labor, and temporary or short-term employment contracts [1,2,3]. Considering not only that maintaining a stable job is very important for employees’ lives but also that the employees function as a critical resource to a firm, the issue of job insecurity is very important for both the employees and the company. To adequately deal with the issue, many scholars and practitioners have paid much attention to the job insecurity in an organization.

Job insecurity means “the anticipation of this stressful event in such a way that the nature and continued existence of one’s job are perceived to be at risk” (p. 27) [2]. Scholars have found that the fundamental feature of job insecurity is the degree of uncertainty about an employee’s job continuity [4]. As one of the most serious job stressors, job insecurity has been known to be related to a member’s poor mental/physical well-being, poor work attitudes/behavior (e.g., job satisfaction, organizational commitment, job involvement, trust in the organization, and organizational citizenship behavior), and poor organizational performance [1,2,3,5,6,7,8,9,10,11].

Although many previous works have investigated the influence of job insecurity on various organizational outcomes including employees’ perception, attitudes, and behaviors as well as performance, as described above, I believe that there have been some research gaps that need to be dealt with [11]. First, extant studies on the association between job insecurity and various organizational outcomes have reported inconclusive results [3,11]. Some studies which are based on both stress-related mechanism [6,12] and social exchange-related mechanism [13,14] have reported that job insecurity functions as a substantial detrimental factor to the work attitudes, well-being, and performance of employees [5,8]. However, other studies have shown that job insecurity is not associated with performance [2,15,16]. In addition, some works which relied on a job-preservation motivation mechanism have suggested that job insecurity can motivate employees to conduct their job better for securing it [16,17]. These mixed results are closely related to the lack of studies which investigate the intermediating mechanisms between job insecurity and organizational outcomes [11]. Therefore, I need to pay more attention to elaborate mechanisms which explain the relationship.

Second, the existing works on the association between job insecurity and organizational outcomes have relatively paid less attention to the employees’ behaviors, mainly concentrating on their attitudes (e.g., job satisfaction, organizational commitment, and organizational identification) or physical/mental health [11]. Although the attitudes or physical/health of employees are important indicators in an organization, the behaviors of members function as the direct results or consequences of the indicators as well as the antecedents of a variety of organizational outcomes (e.g., task performance, turnover intentions, and absenteeism). In addition, to the best of my knowledge, previous studies on the relationship between job insecurity and employee’s behaviors have focused on the bivariate relationship of the variables. For example, Reisel and his colleagues [18] reported that job insecurity is negatively related to the organizational citizenship behavior of employees. Also, another study showed that there is a U-shaped association between job insecurity and organizational citizenship behavior [1]. Thus, there have been few studies which investigate the elaborate intermediating processes (i.e., mediators) of the association.

Lastly, there are methodological issues which should be dealt with. According to a review paper by Shoss [11], previous studies have tended to utilize cross-sectional data in analyzing its hypotheses [14,19,20,21,22]. To elaborately investigate the intermediating mechanisms of the relationship between job insecurity and organizational outcomes, a longitudinal approach rather than a cross-sectional method is requested. In addition, existing works have relied on self-report surveys to measure each employee’s organizational citizenship behavior. However, considering that the self-report method is not free from various biases (e.g., common method variance, social desirability bias, and implicit theories), other ways to measure the variable are needed.

To adequately deal with the research gaps, this paper investigates the underlying mechanisms between job insecurity and organizational citizenship behavior. Organizational citizenship behavior means an individual employee’s behavior, which is discretionary, not officially described by the formal rules or systems, and which facilitates the efficiency of the operation of the organization [23]. Previous research has reported that this variable tends to build the organizational, social, and psychological factors which function as the “catalyst for work processes of the organization” [24]. Although some works have delved into the association between job insecurity and organizational citizenship behavior, to the best of my knowledge, those did not examine the elaborate underlying processes of the association [11]. Thus, this study investigates the intermediating process of it.

In specific, this paper suggests that organizational trust and organizational identification would sequentially mediate the relationship between job insecurity and organizational citizenship behavior. Organizational trust has been considered as a critical work-related attitude as well as one of the most important antecedents of various organizational outcomes [25,26,27,28,29,30]. Grounded on an influential model of the trust and social exchange theory, extant works have shown that job insecurity decreases the level of an employee’s organizational trust [5,31,32].

In addition, prior studies have reported that organizational trust is positively associated with an employee’s positive workplace attitudes, such as organizational identification [33,34,35,36]. Organizational identification is defined as “the perception of oneness with or belongingness to the organization” (p. 22) [37]. Organizational identification is considered as a root construct in that it substantially contributes to creating a strong bond between members and their organization [38]. In terms of the relationship between organizational trust and identification, trust functions as a fundamental social glue which cultivates member’s long-term attachment to an organization [39]. In other words, the feeling of trust in his or her organization is a foundation for their cognitive and affective responses towards the organization, such as organizational identification [40]. Then, this study expects that organizational identification would influence organizational citizenship behavior. This argument is supported by many existing works which demonstrated that organizational identification is an important predictor of organizational citizenship behavior [41,42,43].

To integrate the arguments based on overarching theory, this paper grounds on a context–attitude–behavior framework [44,45], which supports my sequential mediation structure. The framework suggests that a variety of contexts in an organization (e.g., systems, rules, and environments) functions as an initiator that substantially influences the attitudes and behaviors of members. Relying on this, I suggest that job insecurity reflects the contexts that build their behavior (i.e., organizational citizenship behavior) through influencing their attitudes (i.e., organizational trust and organizational identification).

## 2. Theories and Hypotheses

### 2.1. Job Insecurity and Organizational Trust

Previous studies have reported that an employee’s job insecurity decreases the level of his or her organizational trust [5,31,32]. Although the studies empirically demonstrated the association between job insecurity and organizational trust, those did not provide enough theoretical explanations on the relationship [11]. Thus, in this paper, I suggest two theoretical perspectives (i.e., influential model of trust and social exchange theory) to elaborately describe it. 

First, the relationship between job insecurity and organizational trust can be supported by the influential model of trust, which suggest that ability, benevolence, and integrity function as critical factors to form the trust of a trustor in a trustee [27,30]. Ability means the skills, knowledge, or capacities that allow a trustee to have an influence on a trustor in a specific area; benevolence indicates “the extent to which a trustee is believed to want to do good to the trustor, aside from an egocentric profit motive” (p. 718) [27]. Finally, integrity “involves the trustor’s perception that the trustee adheres to a set of principles that the trustor finds acceptable” (p. 719) [27]. If an organization as a trustee possesses the characteristics, an employee of the organization as a trustor would perceive that his or her organization is trustworthy. By applying the influential model of trust into job insecurity research, I can expect that job insecurity may decrease the degree of the organizational trust of employees because job insecurity cannot fulfill the three critical criteria to form trust. 

Specifically, when an employee of an organization feels that his or her job is not secure, the employee may perceive that the organization does not have enough capacities or resources to maintain and support the employee. He or she is likely to acknowledge that only firms with sufficient economic and social resources can keep its employees’ jobs safely. Thus, the employee’s skeptical perceptions on the capabilities of the organization which originated in job insecurity would indicate that the organization cannot fulfil the criterion of ability. Next, job insecurity cannot satisfy the criterion of benevolence. From the perspective of employees, their job’s instability from various events in an organization (e.g., downsizing and layoffs) is not for their own interests but for their organization itself. In other words, job insecurity not only implies the firm’s egocentric profit motive but also is not in the least benevolent to employees. Lastly, when employees feel that their job is insecure, they may perceive that their organization does not have an adequate level of integrity. Although the fundamental purposes of firms are wide and broad, it is very difficult to deny that one of the most important objectives of firms is social contributions. Hiring and maintaining employees are one of the critical contributions of firms [46,47]. Thus, job insecurity may make employees perceive that their firm does not pursue social values. Eventually, the integrity criterion cannot be satisfied.

Second, the influence of jog insecurity on organizational trust can be explained by a social exchange perspective [29]. Job insecurity can build a social exchange mechanism between employees and their organization, directly influencing their attitudes such as organizational trust [11,13,14]. A social exchange perspective relies on the principle of reciprocity. In an association which is based on a social exchange process, when one group gives a benefit to other group, the recipient group may perceive a sense of obligation which they have to repay with something worthy of it [29]. The principle of reciprocity may be applied into job insecurity research; job instability is one of the most serious problems that infringes on employees’ interests. Therefore, when employees feel that their job is not secure, they may perceive a psychological contract breach in terms of the relationship between themselves and their organization. Then, they cannot have a sense of responsibility that they should repay. They may even feel that they should repay the uncomfortable feelings which originated in the job insecurity with negative behaviors towards the organization.

The decreased or even deteriorated sense of duty to repay a firm would worsen employees’ perceptions and attitudes toward the firm in a negative way. From the perspective of employees, one of the available methods to negatively repay these feelings is to form negative attitudes toward the firm in the form of a low level of organizational trust or distrust [11,13,14]. By possessing the negative attitudes, employees can balance the psychological breach due to job insecurity with their organization. Thus, I propose the following hypothesis.

**Hypothesis** **1:** *Job insecurity is negatively associated with employee’s organizational trust.*

### 2.2. Organizational Trust and Organizational Identification

Prior research supports a link between organizational trust and a member’s positive workplace attitudes, such as organizational identification [33,34,35,36]. Through organizational identification, members perceive themselves as psychologically connected with the fate of their organization [48]. Leading to convergent expectations among members, it motivates them to coordinate their efforts to achieve the goals of the organization by improving the quality of their relationships and the cooperation in the organization [49].

Moreover, members who identified with their organizations were more likely to adopt the organizational goals as their own goals and to concentrate on tasks which benefited the organizations beyond their self-interest [37,38], resulting in positive outcomes in the organization, such as cooperative behavior, extra-role behaviors, and intention to stay with their organization [48,50].

Specifically, the social exchange theory [29] provides theoretical mechanisms which link organizational trust and organization identification. According to this perspective, employees who perceive organizational trust are likely to repay the organization with positive attitudes towards the organization (e.g., organizational identification).

The trust of employees toward their organization may lead them to experience a high-quality exchange relationship with their organization and its representatives (i.e., coworkers and leaders) [36]. The positive experience that originated in the trust, in turn, leads members to cultivate shared feelings of responsibility and obligation to the organization as a whole [34,35]. From their point of view, one of the fundamental ways to reciprocate the favorable treatment of organization is through enhancing the positive attitudes toward the organization [33]. These positive work attitudes are likely to encourage them to identify with the organization strongly [36,40]. Therefore, I posit this hypothesis.

**Hypothesis** **2:** *Employee’s organizational trust is positively associated with his or her organizational identification.*

### 2.3. Organizational Identification and Organizational Citizenship Behavior

Many existing works have reported that organizational identification is an important predictor of organizational citizenship behavior [41,42,43]. In this research, based on previous studies, I define organizational citizenship behavior as “any discretionary individual extra-role behavior advantageous to the organization” (p. 284 [43], p. 3 [51]). The existing works have demonstrated that organizational citizenship behavior is related to both individual-level organizational performance (e.g., employee performance, turnover intentions, and absenteeism) and organizational-level performances (e.g., productivity and customer satisfaction) [52,53,54].

Organizational identification is likely to increase an employee’s voluntary behaviors which are not official obligations pertinent to his or her tasks despite the fact that the actions would not be rewarded by the organization [55]. For example, employees who strongly identify themselves with their organization are likely to voluntarily help their organization to pursue its collective goals beyond their private interests, since they feel as if the objectives of the organization are their own [42,43]. From the perspective of the strong identifiers, helping colleagues in their organization by conducting organizational citizenship behavior is very compatible with helping themselves because the colleagues play a meaningful role to define their selves [37,38,42,43]. They tend to define themselves by relying on a collective identity which originated in the organization, as a “good citizen” [46]. Therefore, I posit this hypothesis.

**Hypothesis** **3:** *An employee’s organizational identification is positively associated with his or her organizational citizenship behavior.*

### 2.4. Sequential Mediating Role of Organizational Trust and Organizational Identification between Job Insecurity and Organizational Citizenship Behavior

**Figure 1:**
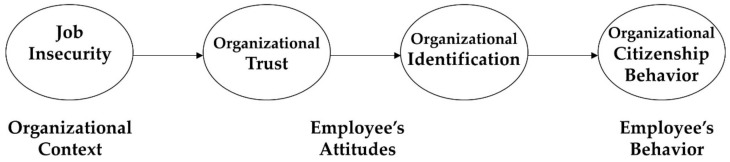
The framework of the research model.

As described above, this paper suggests that organizational trust and organizational identification sequentially mediate the relationship between job insecurity and organizational citizenship behavior. According to the above arguments, I believe that an employee’s job insecurity decreases the level of his or her organizational citizenship behavior through negatively influencing his or her level of organizational trust and organizational identification. To adequately integrate the above hypotheses from the perspective of theoretical soundness, I draw on a context–attitude–behavior framework [44,45], which supports my sequential mediation structure. The framework suggests that various contexts in an organization (e.g., systems, rules, and environments) function as an initiator that significantly affects attitudes and behaviors of members. Based on it, I expect that job insecurity reflects the contexts that build their behavior (i.e., organizational citizenship behavior) through influencing their attitudes (i.e., organizational trust and organizational identification). Extant works theoretically and empirically support my hypotheses by showing a positive association between job insecurity and organizational trust [5,31,32], between organizational trust and organizational identification [33,34,35,36], and between organizational identification and organizational citizenship behavior [41,42,43]. Thus, I hypothesize the following (please see Figure 1).

**Hypothesis** **4:** *An employee’s organizational trust and organizational identification sequentially mediate the relationship between job insecurity and organizational citizenship behavior.*

## 3. Method

### 3.1. Data Collection

By utilizing an online survey method, this paper gathers data from currently working South Korean employees with three different time points. One of the largest online research companies in South Korea (i.e., having about 1,300,000 panelists) implemented the procedures. The research company randomly chose the participants to decrease the possibility of sampling bias. Through the process, the bias from various employee’s characteristics which may influence the results of this paper (e.g., gender, tenure, position, education, and industry type) would be reduced. Due to its various operating functions in the online systems, this paper was able to track down who responded to it, implying that respondents from time point one to time point three are the same.

At time point one, a total of 512 employees participated in my survey. At time point two, 378 employees responded to the second survey. Lastly, at time point three, 335 employees responded to the third survey. The time span between each time point was 4 weeks. After gathering the data, I eliminated any missing data. The final data was from 303 respondents. The characteristics of the sample are described below (please see Table 1).

### 3.2. Measures

This study measures the study variables with a five-point Likert scale (1 = strongly disagree, 5 = strongly agree). Then, this research computed the internal consistency of the variables by utilizing Cronbach alpha values.

#### 3.2.1. Job Insecurity (Time Point 1, Gathered from Employees)

To measure the degree of job insecurity, I utilized four items of the job security scale from Kraimer and his colleagues [56]. The sample items were “If my current organization were facing economic problems, my job would be the first to go.”; “I will not be able to keep my present job as long as I wish.”; “My job is not a secure one.”; and “My job will not be there although I want it.” The value of Cronbach alpha in this research was 0.87.

#### 3.2.2. Organizational trust (Time Point 2, Gathered from Employees)

Three items were used for organizational trust from previous research [57]. Considering the suggestion of the paper [57], the core items of the measure were chosen. The sample items were “I trust my organization.” and “I feel that my company is reliable.” The value of Cronbach alpha in this research was 0.95.

#### 3.2.3. Organizational Identification (Time point 3, Gathered from Employees)

This paper utilized four items of organizational identification measure which was used in a previous work [48]. The sample items were “It is as if I were insulted if someone criticizes the company I work for.”; “My company’s success ultimately means my own success.”; and “I usually say ‘our’ company when talking about my company.” The value of Cronbach alpha in this study was 0.83.

#### 3.2.4. Organizational Citizenship Behavior (Time Point 3, Gathered from an Immediate Leader of Each Employee)

To measure the degree of an employee’s organizational citizenship behavior, an immediate leader of the employees rated it. I used five items of organizational citizenship behavior scale from the existing research [58]. The sample items were “This employee helped a coworker who had too much to do.”; “This employee helped new employees get oriented to the job.”; and “This employee lent a compassionate ear when someone had a work problem.” The value of Cronbach alpha in this study was 0.90. I believe that gathering data from multiple sources may diminish the potential issue of a common method bias.

#### 3.2.5. Control Variables

Variables were included to the control factors which influenced organizational citizenship behavior. As control variables, I utilized gender, position, tenure, and education level [59,60]. The variables were gathered at time point 2.

### 3.3. Statistical Analysis

To analyze the data, this study conducted a correlation analysis. Then, because the research model included various variables, this paper utilized structural equation modeling (SEM) to analyze the sequential mediation model and to obtain the fit indices [61]. Considering the suggestion of Anderson and Gerbing [62], I took a two-step approach, which included the measurement model and the structural model. To evaluate the adequacy of the model fit, I considered several goodness-of-fit indices. Adequate fit indices indicated a comparative fit index (CFI) and a Tucker-Lewis index (TLI) greater than 0.90, and a root mean square error of approximation (RMSEA) less than or equal to 0.06 [63].

## 4. Results

### 4.1. Descriptive Statistics

The results of the descriptive analysis are described in Table 2. The main variables including independent variable, mediators, and dependent variable are highly correlated.

### 4.2. Measurement Model

To check whether there is an adequate level of discriminant validity, this paper conducted a confirmatory factor analysis (CFA) for the study variables from a same employee (i.e., job insecurity, organizational trust, and organizational identification). In this CFA, organizational citizenship behavior was not included because the variable was gathered from each employee’ immediate leader, not the employee him/herself. The three-factor model has a good fit to the observations (χ^2^ (d*f* = 49) = 101.37; CFI = 0.977; TLI = 0.969; RMSEA= 0.059). Then, I conduct sequential chi-square (χ^2^) difference tests to compare the three-factor model with the two-factor and single-factor models. The results of the test showed that the three-factor model had the best fit among all the alternative models (please see Table 3). Therefore, I confirmed the research variables would be distinctive.

### 4.3. Structural Model

This study established a sequential mediation model using the SEM technique. In this structure, the relationship between job insecurity and organizational citizenship behavior is sequentially mediated by organizational trust and organizational identification.

**Figure 2:**
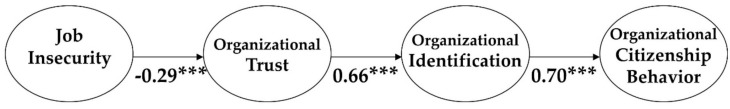
The standardized estimate values of the final model. Notes: *** *p* < 0.001.

To check whether job insecurity directly or indirectly influences organizational citizenship behavior, this paper compared my hypothetical model (i.e., full mediation model) to an alternative nested model (i.e., partial mediation model) by conducting a chi-square difference test [64]. The fit indices of the full mediation model (Model 1) was adequate: χ^2^ = 323.45 (d*f* = 176), CFI = 0.957, TLI = 0.948, and RMSEA = 0.053. Also, the alternative nested model (Model 2) includes a direct path from job insecurity to organizational citizenship behavior. Although the fit indices of Model 2 are also adequate (χ^2^ = 321.72 (d*f* = 175); CFI = 0.957; TLI = 0.948; RMSEA = 0.053), the result of chi-square difference test between Model 1 and Model 2 shows that Model 1 was better than Model 2 (Δχ^2^ (1) = 1.73, nonsignificant). In the final model, all the control variables (i.e., position, gender, tenure, and education level) were not statistically significant. The model demonstrated that job insecurity is significantly and negatively associated with organizational trust (*β* = −0.29, *p* < 0.001), and organizational trust was positively related to organizational identification (*β* = 0.66, *p* < 0.001). Lastly, organizational identification was positively associated with organizational citizenship behavior (*β* = 0.70, *p* < 0.001). The results suggest that Hypothesis 1, 2, and 3 were supported (please see Figure 2).

### 4.4. Bootstrapping

This paper conducted a bootstrapping analysis with a sample of 5000 to check whether the Hypothesis 4 which expects that there is a sequential indirect effect between job insecurity and organizational citizenship behavior is correct. Please consider that the indirect mediation effect is significant at a 5% level when the 95% bias-corrected confidence interval (CI) for the mean indirect mediation effect does not include zero [65]. In this analysis, the bias-corrected CI for the effect on the pathway from job insecurity via organizational trust and organizational identification to organizational citizenship behavior excluded zero (95% CI = [−0.20, −0.08]). Thus, the result implies that the sequential indirect mediation effect of organizational trust and organizational identification on the path is significant at a level of 5%, supporting Hypothesis 4.

## 5. Discussion

This paper delved into the underlying mechanism of the association between job insecurity and organizational citizenship behavior. To empirically test the above hypotheses, 3-wave time-lagged data from members in organizations of South Korea were used. By conducting a sequential mediation model analysis using the SEM method, I demonstrated that an employee’s organizational trust and organizational identification sequentially mediated the association between job insecurity and organizational citizenship behavior. In this section, I discuss the finding’s theoretical and practical implications, as well as the limitations and suggestions for future research.

### 5.1. Theoretical Implications

This study may contribute to extending the job insecurity literature by providing theoretical implications. First, by investigating the intermediating mechanisms between job insecurity and organizational outcomes with a 3-wave time-lagged dataset from employees in South Korean firms, this paper may contribute to resolving the mixed results between the variables [11]. Extant studies on the association between the variables have reported inconclusive results [3,11]. Some studies have shown that job insecurity decreases the quality of work attitudes, well-being, and performance of employees [5,8]. However, other studies have shown that job insecurity is not related to performance [2,15,16]. Moreover, some studies have suggested that job insecurity can motivate employees to conduct their jobs better to secure their jobs [16,17]. In this paper, by empirically testing the elaborate mechanism of the relationship with a 3-wave time-lagged dataset from employees in South Korean firms, I demonstrated that job insecurity would decrease the level of organizational citizenship behavior through deteriorating the quality of an employee’s organizational trust and identification. This result means that job insecurity has harmful effects on employee’s attitudes (i.e., organizational trust and organizational identification) as well as his or her behaviors (i.e., organizational citizenship behavior), thus, eventually diminishing organizational performance. I believe that these results may contribute to resolving the mixed results of the relationship.

Second, in this research, this paper examined the influence of job insecurity on an employee’s behavior, especially, organizational citizenship behavior. Considering not only that the extant works on the job insecurity-organizational outcome link have paid relatively less attention to employees’ behaviors [11] but also the behaviors of employees function as antecedents of a variety of organizational outcomes (e.g., task performance, turnover intentions, and absenteeism), investigating the impact of job insecurity on employee’s behaviors is meaningful. Moreover, by examining the sequential mediating mechanisms between job insecurity and organizational citizenship behavior, I demonstrated that job insecurity tends to negatively influence employees’ organizational citizenship behavior beyond the existing mixed results as described above through decreasing the quality of their organizational trust and identification. This would contribute to elaborating the previous literature on the relationship.

Lastly, I believe that this research may contribute to dealing with methodological issues. By utilizing a 3-wave time lagged dataset in conducting the structural equation modeling (SEM) analysis, this paper may complement the limitations of a cross-sectional approach that previous studies have taken. In addition, in the present research, I measured an employee’s organizational citizenship behavior by utilizing an evaluation of the employee’s immediate leader, beyond the self-report approach. I believe that these would contribute to adequately dealing with the methodological gaps which the extant works had (e.g., common method variance, social desirability bias, and implicit theories).

### 5.2. Practical Implications

I believe that the findings of this paper can provide business leaders with practical insights. First, through this paper, I emphasize the importance of employees’ job insecurity in an organization by testing that it decreases the quality of their organizational citizenship behavior. Considering that organizational citizenship behavior can be considered as an indicator which judges whether an organization including its employees, climates, and systems is operating in an adequate and effective way [54], leaders or top management teams should seriously acknowledge the harmful effects of job insecurity on organization. Moreover, beyond just acknowledgement, they have to monitor and intervene to adequately deal with the negative influences of job insecurity. Specifically, top management teams and leaders in an organization should find ways to decrease an employee’s job insecurity. For example, if an organization establishes formal systems or rules which stabilize employees’ contracts of employments, the employees would show a greater level of organizational citizenship behavior.

Second, this paper may be helpful for leaders or top management teams who attempt to decrease the negative impacts of employees’ job insecurity. When the leaders or top management teams begin to operate various practices or systems for dealing with the harmful effects, they may feel that they need to have criteria for judging whether the practices and systems function well. In that situation, the finding that job insecurity decreases the quality of the behavior through deteriorating the level of employee’s organizational trust and identification can provide the criteria. For example, when the practices or systems for decreasing the negative effects of job insecurity are effectively operated, the level of employee’s trust and identification would be enhanced due to the positive influences of the practices or systems. Thus, leaders and top management teams can check the effectiveness of their attempts through observing the level of those variables (i.e., organizational trust and identification).

### 5.3. Limitations and Suggestions for Future Research

Despite of its theoretical and practical implications, this paper has some limitations that should be dealt with. First, in this research, I could not consider the objective external factors which can influence an employee’s job insecurity. The job insecurity variable in this paper is measured as a subjective perception of each employee. Although the perception can reflect the objective possibility of losing a job, I believe that the perceptions on job insecurity should be controlled by objective external factors in an organization such as downsizing rate, promotion rate, overall characteristics of human resources management systems, and the level of social insecurity systems. In future research, scholars should consider the factors.

Second, although this research utilized a 3-wave time-lagged dataset, strictly speaking, it could not capture the change (i.e., increase or decrease) of the mediating mechanisms. To deal with this issue, by designing a longitudinal research, future studies should reveal how job insecurity would increase or decrease an employee’s organizational trust and then organizational identification, eventually influencing his or her organizational citizenship behavior.

Third, pertinent to the first limitation described above, I could not consider the differences in the objective external factors which affect employee’s job insecurity. In this paper, I obtained data from firms only from South Korea. However, considering that the external factors are likely to vary from country to country and from culture to culture. For example, in the Scandinavian countries (e.g., Sweden, Norway, and Denmark) which have a high-level of social security systems, the impacts of job insecurity on an employee’s working life are relatively less serious than countries which have a low-level of the systems. Future studies should better consider this issue.

## 6. Conclusions

Although the research has some limitations, by utilizing a 3-wave time-lagged dataset with a structural equation modeling (SEM) analysis, this research found that an employee’s organizational trust and identification sequentially mediated the relationship between job insecurity and organizational citizenship behavior. I expected that it would contribute to job insecurity literature by delving into elaborate underlying mechanisms between job insecurity and organizational citizenship behavior. The findings of this study suggest that an employee’s trust in his or her organization and identification with it function as intermediating processes which explain the relationship. I believe that it has theoretical and practical implications for the literature.

## Figures and Tables

**Table 1:** Descriptive features of the sample.

Characteristic	Percent
Gender	
Male	47.2%
Female	52.8%
Age	
20s	21.8%
30s	24.1%
40s	27.0%
50s	27.1%
Occupation	
Office workers	64.0%
Administrative positions	19.5%
Sales and marketing	6.6%
Manufacturing worker	4.3%
Education	1.3%
Position	
Staff	29.0%
Assistant manager	25.1%
Manager or deputy general manager	30.4%
Department/general manager and above director	15.5%
Tenure (in month)	
Below 50	51.8%
50 to 100	19.1%
100 to 150	14.6%
150 to 200	5.2%
200 to 250	4.3%
Above 250	5.0%
Firm size	
Above 500 members	17.5%
300–499 members	5.6%
100–299 members	16.8%
50–99 members	13.5%
Below 50 members	46.5%
Industry Type	
Manufacturing	26.1%
Services	14.9%
Construction	13.5%
Information service and telecommunications	9.9%
Education	10.0%
Health and welfare	8.3%
Public service and administration	7.3%
Financial/insurance	4.0%

**Table 2:** The means, standard deviations, and correlations between the variables.

Variable	Mean	SD	1	2	3	4	5	6	7
1. Gender_T2	1.53	0.50	-						
2. Position_T2	2.56	1.39	−0.36 **	-					
3. Tenure (Months)_T2	78.04	79.00	−0.12 *	0.32 **	-				
4. Education_T2	2.58	0.81	−0.07	0.16 **	0.01	-			
5. Job insecurity_T1	3.12	0.80	−0.03	0.07	-0.07	0.11	-		
6. OT_T2	3.01	0.82	0.01	0.13 *	0.11	−0.10	−0.29 **	-	
7. OI _T3	3.34	0.66	0.04	0.22 **	0.18 **	0.04	−0.17 **	0.57 **	-
8. OCB_T3	3.15	0.69	0.08	0.07	0.12 *	−0.03	−0.18 **	0.53 **	0.58 **

Note: * *p* < 0.05. ** *p* < 0.01. OT, OI, and OCB mean organizational trust, organizational identification, and organizational citizenship behavior, respectively. As for gender, males are coded as 1 and females are coded as 2. As for position, general manager or higher are coded as 5, deputy general manager and department manager are coded as 4, assistant manager is coded as 3, clerk is coded as 2, and others below clerk are coded as 1. As for education, the “below high school diploma” level is coded as 1, the “community college” level is coded as 2, the “bachelor’s” level as coded as 3, and the “master’s degree or more” level is coded as 5.

**Table 3:** The chi-square difference tests among the alternative measurement models.

Model	χ^2^	*df*	CFI	TLI	RMSEA	Δ*df*	Δχ^2^	Preference
1-Factor Model	859.02	52	0.641	0.544	0.227			
2-Factor Model that integrates organizational trust and identification	353.92	51	0.865	0.826	0.140	1	505.10	2-Factor Model
3-Factor Model	101.37	49	0.977	0.969	0.059	2	252.55	3-Factor Model
